# Influence of host nutritional condition on post-infection traits in the association between the manipulative acanthocephalan *Pomphorhynchus laevis* and the amphipod *Gammarus pulex*

**DOI:** 10.1186/s13071-015-1017-9

**Published:** 2015-07-30

**Authors:** Sophie Labaude, Frank Cézilly, Xavier Tercier, Thierry Rigaud

**Affiliations:** Université de Bourgogne Franche-Comté, UMR CNRS 6282 Biogéosciences, Dijon, France

**Keywords:** Deprivation, Energetic constraints, Food resources, *Gammarus pulex*, Parasite manipulation, *Pomphorhynchus laevis*

## Abstract

**Background:**

Several parasites with complex life-cycles induce phenotypic alterations in their intermediate hosts. According to the host manipulation hypothesis, such phenotypic alterations are supposed to increase the fitness of the parasite at the expense of that of its intermediate hosts through increasing the probability of transmission to next hosts. Although the phenomenon has received a large attention, the proximate factors modulating the occurrence and intensity of host manipulation remain poorly known. It has however, been suggested that the amount of energy reserves in the intermediate host might be a key parameter, although its precise influence on the intensity of manipulation remains unclear. Dietary depletion in the host may also lead to compromise with other parasite traits, such as probability of establishing or growth or virulence.

**Methods:**

Here, we address the question through performing experimental infections of the freshwater amphipod *Gammarus pulex* with two different populations of the acanthocephalan fish parasite *Pomphorhynchus laevis*, and manipulation of host nutritional condition. Following exposure, gammarids were given either a “standard” diet (consisting of elm leaves and chironomid larvae) or a “deprived” food treatment (deprived in proteins), and infection parameters were recorded. Once parasites reached the stage at which they become infective to their definitive host, refuge use (a behavioural trait presumably implied in trophic transmission) was assessed, and metabolic rate was measured.

**Results:**

Infected gammarids exposed to the deprived food treatment showed a lower metabolic rate, indicative of a lower body condition, compared to those exposed to the standard food treatment. Parasite size was smaller, and, depending on the population of origin of the parasites, intensity of infection was lower or mortality was higher in deprived hosts. However, food treatment had no effect on either the timing or intensity of behavioural modifications.

**Conclusions:**

Overall, while our results suggest that acanthocephalan parasites develop better in hosts in good condition, no evidence was found for an influence of host nutritional condition on host manipulation by parasites.

## Background

Many parasites with complex life cycles are known to alter the phenotype of their hosts [1, 2]. In particular, trophically-transmitted parasites often induce phenotype modifications in their intermediate hosts that appear to make them more vulnerable to predation by definitive host species, thus possibly increasing their probability of completing their life cycle [3] (but see [4, 5]). This phenomenon, known as “parasite manipulation”, has been shown to play diverse and important roles, such as altering host population ecology [6], affecting food webs in ecosystems [7], or driving disease dynamics [8]. However, and despite numerous examples of behavioural alterations in many different host-parasite associations [2], this phenomenon is not yet fully understood [9–11].

In particular, the proximate factors that modulate the occurrence and intensity of host manipulation remain poorly known. It has however, been suggested that the amount of host’s energy reserves could play a key role, although its precise influence remains unclear. On the one hand, it has been predicted that parasites should adjust their exploitation strategy to the physiological condition of their hosts, possibly leading to an increase or acceleration of behavioural changes in hosts in poor condition [12]. This is because the risk for a parasite to die before trophic transmission occurs should be higher in hosts in poor nutritional condition [13, 14]. On the other hand, it has been suggested that displaying a modified behaviour is costly for hosts, such that only hosts in good body condition should be able to show altered behaviour [15]. More recently, Maure *et al.* [16] suggested that parasites have been selected to leave enough resources to their hosts to allow them to express manipulated behaviours (the “host energetic resource constraint hypothesis”, hereafter HERC hypothesis).

So far, only a few studies have addressed the importance of energy resources in the interaction between manipulative parasites and their hosts. However, some evidence exists for an energetic cost of harbouring a manipulative parasite. For instance, Lettini and Sukhdeo [17] showed that isopods infected by acanthocephalan parasites allocated about 21 % of their energy production to parasite growth, at the expense of their own reproduction (but see [18]). Other studies have provided some evidence for reduced growth resulting from competition for host resources in manipulative parasites co-occurring in a single host [19], or have revealed negative associations between the speed of parasite development and the intensity of manipulation [20], or between host survival and parasite fecundity [21]. All those studies tend to suggest that host energetic reserves are a limited resource for the parasite. In addition, it has been shown that host resources could be modified by the presence of a parasite. In particular, glycogen content was increased in the amphipod *G. pulex* [22] and in the isopod *Caecidotea intermedius* [23] infected by acanthocephalan parasites, compared to uninfected individuals, while additional modifications in lipid and glucose contents was also observed in the amphipod *Gammarus insensibilis* infected by a trematode parasite [6].

Although the potential influence of host resources on the interaction between hosts and manipulative parasites has been emphasized, no study so far has directly addressed the question. Here, we experimentally tested the HERC hypothesis [16] using one of the most studied systems in parasite manipulation, the acanthocephalan parasite *Pomphorhynchus laevis* and its intermediate host, the freshwater crustacean amphipod *Gammarus pulex* [24]. This parasite reproduces in different fish species and grows in its intermediate gammarid host, inducing, once the infective larval stage (cystacanth stage) has been reached, numerous behavioural alterations, such as reversed reaction to light [25], decreased conspecific attraction [26] or reduced refuge use [27], the latter being linked with the probability of predation by definitive fish hosts [27–29].

We relied on experimental infections of *G. pulex* collected in the wild to address the influence of host nutritional condition on the intensity of manipulation and classical infection parameters such as prevalence and intensity. To test for the effect of resources, we provided gammarids with either a standard or a deprived food treatment during parasite development. We then followed infection parameters (survival of gammarids, infection prevalence and intensity, developmental stage of parasites), assessed metabolism, and performed behavioural tests on both infected and control gammarids. We measured a single behaviour only, the rate of refuge use, because, although *P. laevis* induces an infection syndrome in its host (i.e. a series of symptoms that appear to result from some major physiological disruption in the infected host [30]), this is the one which is most directly involved in parasite trophic transmission [28, 29]. According to the HERC hypothesis, lowered host condition should result in a lower exploitation by parasites, possibly an increase in host mortality, and a change in the intensity of behavioural alterations, either in the sense of a decrease, indicative of an unaffordable high cost of performing altered behaviours (*sensu* Thomas *et al.* [15]), or in that of an increase, indicative of a minimization of the risk of premature host death (*sensu* Thomas *et al.* [12]).

## Methods

### Sampling

Uninfected gammarids were collected in a small tributary of the Suzon river (Burgundy, eastern France; 47° 24’12.6”N, 4°52’58.2”E), in October and December 2013. Only males were kept, because parasites fail to develop in females more often than in males [31], whereas sex appears to have generally no effect on the extent of behavioural modifications [25, 32, 33]. Genetic analysis (see [34]), performed on one third of the individuals (n = 330), showed that about 97 % belonged to the species *Gammarus pulex*, with the remaining 3 % belonging to the closely-related *G. fossarum*. Gammarids were acclimated in the laboratory for two days before experimental infections, in a room maintained at 10 °C, which corresponds to the temperature of their natural habitat, and under a 12:12 light:dark cycle.

Naturally infected chubs, *Leuciscus cephalus*, were caught in the Vouge river (Burgundy, eastern France, 47°9’34.36” N 5°9’2.50” E) in October, and in the Vair river (Vosges, eastern France, 48°11’44.3”N 5°53’57.3”E) in December 2013. Adult parasites were taken from the intestines of the fish, and characterised by genetic analyses with the method described in Franceschi *et al.* [31]. Only parasite eggs from the species *P. laevis*, collected from 10 females sampled in five fish for the Vouge population, and in 13 females sampled in two fish for the Vair population, were mixed for each population and used for experimental infections.

Infections were therefore made using hosts and parasites that did not co-evolve. However, we have previously used gammarids from Suzon river for experimental infections, so the system is now highly characterized [19, 20, 30, 31, 35]. Notably, infection in gammarids from the Suzon river reflects the infection characteristics of all other gammarid populations tested so far, but they are more sensitive to acanthocephalan infections [35], allowing to optimize the experimental infection rate. In addition, Perrot-Minnot *et al.* [30] showed that syndromes induced by experimental infection using these hosts and parasites from the Vouge river are highly correlated with those of natural infections.

### Experimental infections and treatments

Experimental infections were performed following the procedure detailed in Franceschi *et al.* [31]. Overall, 374 and 301 individuals were exposed to parasite eggs from the Vair population and the Vouge population, respectively (hereafter referred as “Vair-infected” and “Vouge-infected” individuals). Three hundred control individuals were maintained under the same conditions without eggs. After 48 hours of exposure, gammarids were placed in individual crystallizers, and randomly divided into two groups with different food treatments. Food treatments were chosen according to the natural food regime of gammarids and spatial variation in food availability observed in the field. Indeed, several studies have reported that, if given a choice, gammarids will feed on both leaf materials (shredder regime) and prey (predator regime), while cannibalism is often observed when only leaves are provided [36, 37]. In temperate streams or rivers, the quality and quantity of food resources are highly dependent on environmental factors and, therefore, vary between rivers [38]. Even along the upstream-downstream gradients, both the proportion of leaf detritus and prey availability can vary (e.g. [39, 40]). Thus, individuals from the “standard food treatment” were fed weekly, alternatively with conditioned elm leaves and dead chironomid larvae (which provide a high source of proteins [41]). Individuals from the “deprived food treatment” received only elm leaves, once every two weeks. All individuals were maintained in the same room at 17 °C ± 0.5 with a 12:12 light:dark cycle. Water was changed once every two weeks, using an oxygenated mix of water from the Suzon river and dechlorinated, UV-treated tap water.

### Monitoring

All gammarids were checked on a daily basis. Gammarids found dead were immediately measured and dissected under a binocular microscope to determine the intensity of infection. Although this population of *G. pulex* is not infected by *P. laevis,* individuals can be infected with another acanthocephalan parasite species, *Echinorhynchus truttae*. Such infected individuals (n = 9) were removed from the experiment. Six weeks after infection, all gammarids were checked once a week under a binocular microscope to determine whether they were actually parasitized by *P. laevis*, and to monitor the date of the switch between the acanthella stage (ovoid shape, translucent orange colour) and the cystacanth one (spherical and more pronounced opaque colour [42]). The width of cystacanth larvae from 77 gammarids infected by the Vouge population was measured as a proxy for larval size (n = 160 parasites), in order to determine the effect of food treatment on cystacanth size.

### Behavioural measurements of refuge use

Behaviour was recorded three times (hereafter referred as “rounds”) on all infected individuals: one day, 10 days, and 20 days after the cystacanth stage was detected. Behaviour of control individuals was tested similarly three times. Gammarids were individually placed in boxes (10.5 × 16 cm) filled with 250 mL of water, and labelled with a number, giving no clue about the group treatment to which the gammarids were belonging, and, thus, allowing blind recording. Boxes were containing a refuge at one extremity, consisting of a saucer terracotta pot (8.5 cm of diameter) cut in half, with a one centimetre hole in the convex part (see Dianne *et al.* [43]). A period of 10 minutes of acclimatization was allowed following the introduction of gammarids. Then, the position of each gammarid was recorded every three minutes during 90 minutes, and scores were given for every observation (1 if the individual was inside the refuge, 0 if it was outside), such that summed scores at the end of each round could range from 0 (always outside the refuge) to 31 (always inside).

### Metabolic rate

Metabolic rate was used as a proxy for host body condition, given that it is known to decrease in crustaceans experiencing starvation [44–47]. In addition, acanthocephalan parasites are known to modify various kinds of energetic reserves of their hosts, increasing lipid and glycogen contents, and decreasing glucose content [6, 22, 23]. Relying on metabolic rate can be regarded as a more integrative approach that takes into account all forms of energetic reserves. Metabolic rate was estimated for each gammarid from its oxygen consumption, measured three days after the second round of behaviour measurements (about 13 day-old cystacanths). We used SDR SensorDish® Reader (PreSens, Germany), a non-invasive device based on fluorescence [48], following the protocol presented in Perrot-Minnot *et al.* [30]. As oxygen consumption is known to vary with body mass [49], individuals were weighed immediately after the measure, following a quick drying on soft tissue.

### Statistical analysis

We used a nominal logistic regression to investigate which parameters had an effect on prevalence (i.e. the proportion of gammarids harbouring at least one parasite among those exposed to the infection), and a generalized linear model with a quasi-Poisson distribution and a log link-function to analyse infection intensity among infected individuals. A linear model was used to analyse the effects of food treatment, intensity, and host body size on the size of cystacanth larvae, with individual host identity as a random factor. The speed of parasites development was analysed using chi-square tests.

Survival analysis started on the 39th day after exposure, corresponding to the time when parasites had become large enough to be detected upon dissection. This allowed us to distinguish between actually infected individuals and individuals exposed to infection but not successfully infected. Thus, subsequent statistical analyses (survival as well as metabolic rate and behaviour) do not include individuals exposed to infection in which no parasite developed. Cox regressions were used to analyse host survival. First, we took into account all individuals to investigate the effect of infection status (control or infected with each population of parasites) and food treatment (standard vs. deprived). In a second step, we considered only infected individuals to analyse the relative influences of infection intensity (either one, two, or more than two parasites, see Franceschi *et al.* [31]), population of origin of the parasites, and food treatment.

Metabolic rate, expressed in milligram of O_2_ consumed per minute, was log-transformed to meet normality, and analysed with ANOVAs. We first investigated among all individuals the effect of their mass, food treatment, infection status and all interactions. Then, the same procedure was used considering only infected individuals, to explore the effect of the population of origin of the parasites.

Scores of refuge use were analysed as repeated measures using the nparLD function, a R software package for nonparametric analyses of right-censured longitudinal data, allowing the decrease in sample size with time due to individuals’ death [50]. Among Vair-infected individuals, no gammarid from the deprived food treatment survived until the third behavioural round. Therefore, the effect of food and infection status (control, Vair-infected or Vouge-infected) along time (rounds of measurements: one day, 10 days and 20 days after parasites reached cystacanth stage) were analysed considering only the first and second rounds of behavioural measurements. Another analysis was conducted using the three behavioural rounds, but considering only individuals from the standard food treatment, allowing us to both analyse changes in behaviour over a longer period of time and assess the effect of the population of origin of parasites. For each analysis, ‘ANOVA-type statistics’ were performed, followed by post-hoc ‘pair-comparisons’ (see Noguchi *et al.* [50] for details).

Spearman tests were used in order to check for the presence of potential trade-offs between metabolic rate or mortality rate, and the intensity of behavioural scores. To that end, we used scores from the second round of behavioural tests, since metabolic rate was measured soon after (three days).

Statistical analyses were performed using JMP version 10.0.0 software (SAS Institute, Cary, NC, U.S.A.) and R version 3.1.1 software (R Foundation for Statistical Computing). For each analysis described above, all factors and their second order interactions were first entered in the models. Except for non-parametric analyses where this procedure was not possible, we then compared the Akaike Information Criterion (AIC) among all of the possible models, and presented that one minimizing the AIC.

## Results

### Infection parameters

The overall nominal logistic regression (χ_2_ = 62.29, d.f. = 4, *P* <0.0001) showed that the success of infection (prevalence) varied widely between populations of parasites (Likelihood Ratio Chi-square, LR-χ_2_ = 54.73, d.f. = 1, *P* <0.0001), ranging from 43.13 % for gammarids exposed to parasites from the Vair, to 70.85 % for gammarids exposed to Vouge parasites. The size of gammarids had a significant positive effect on the probability of infection (LR-χ_2_ = 6.23, d.f. = 1, *P* = 0.01), whereas food treatment had none (LR-χ_2_ = 0.86, d.f. = 1, *P* = 0.35).

**Fig. 1:**
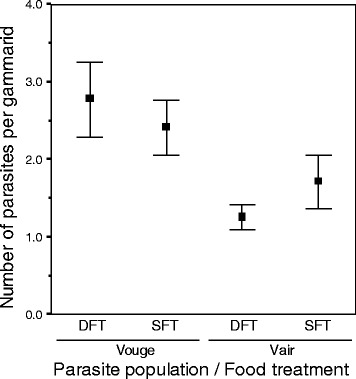
Parasite intensity within a host. Number of parasites per host according to the population of origin of parasites (Vouge and Vair) and the food-treatment received by the host (DFT or SFT, respectively deprived food treatment and standard food treatment). Dots represent means and error bars indicate 95 % confidence interval

Among individuals harbouring parasites, a GLM showed that parasite intensity was significantly influenced by the interaction between food treatment and parasite population (Table 1, Fig. 1). Infection intensity was significantly higher in gammarids infected with parasites from the Vouge population. In this population, the deprived food treatment induced no change in the intensity of infection (χ2 = 1.65, *P* = 0.20), whereas in Vair-infected gammarids infection intensity was lower under the deprived food treatment (χ2 = 4.33, *P* = 0.04) (Fig. 1).

**Table 1:** Effect of parasite population, food treatment and host size on the infection intensity

Source of variation	d.f.	LR-χ2	P
Parasite population	1	52.48	<0.0001
Food treatment	1	1.22	0.27
Gammarids size	1	0.02	0.89
Parasite population x Food treatment	1	8.06	0.004

Whole model: χ_2_ = 57.86, d.f. = 4, *P* <0.0001

Generalized linear model analysing the effect of parasite population, food treatment and gammarid size on the infection intensity (number of parasites harboured by gammarid hosts). A quasi-Poisson error term and a log link-function were used. The model presented here minimized the AIC criterion

**Fig. 2:**
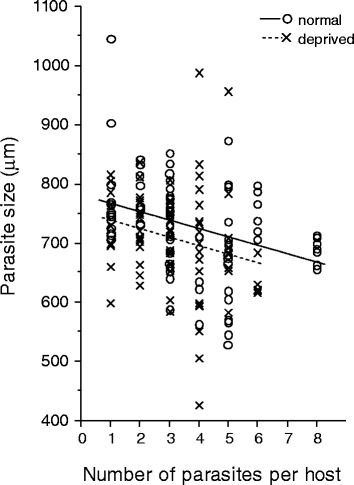
Parasites size at infective stage. Size of cystacanth larvae, according to infection intensity in each host, and food treatment. Each dot represents the width of a larvae (μm). Circles and dotted line stand for the deprived food treatment, while crosses and full line represent the standard food treatment

The width of cystacanth larvae followed a normal distribution. The model minimizing the AIC contained food treatment, parasite intensity and their interaction. The size of cystacanth larvae decreased with infection intensity (F_1, 53.65_ = 8.50, *P* = 0.005). Parasites from the deprived food treatment tended to reach a smaller size than those from the standard food treatment (Fig. 2; F_1, 45.12_ = 2.73, *P* = 0.11), whereas the interaction between food treatment and infection intensity was not significant (F_1, 53.65_ = 0.10, *P* = 0.75), indicating that infection intensity and food treatment had additive effects on parasite size.

Parasites were remarkably homogeneous in their development time, with all cystacanths appearing between the 10th and the 11th week after the infection, regardless of the population considered. In addition, there was no effect of food treatment on the speed of development (Chi-square test: χ_2_ = 0.42, d.f. = 3, *P* = 0.94).

### Host survival

Cox regression (χ_2_ = 100.37, d.f. = 5, *P* <0.0001) considering all individuals showed that survival was significantly influenced by food treatment (LR-χ_2_ = 16.02, d.f. = 1, *P* <0.0001), infection status (infected with each of the two parasite populations, or control individuals; LR-χ_2_ = 67.04, d.f. = 2, *P* <0.0001) and their interaction (LR-χ_2_ = 13.76, d.f. = 2, *P* = 0.001).

**Fig. 3:**
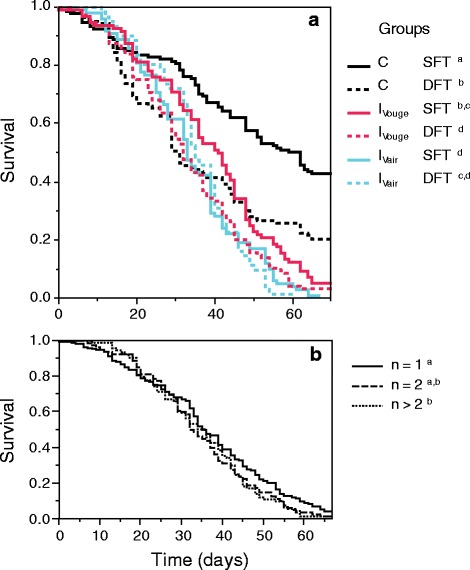
Hosts survival according to infection status and food treatment. Survival curves (**a**) for all gammarids of the experiment according to their status (Control C, or infected by parasites from the Vouge or from the Vair rivers, respectively I_Vouge_ and I_Vair_), and food treatment (standard food treatment SFT or deprived food treatment DFT); and (**b**) for infected gammarids according to the number of parasites they harbour (n = one, two or more than two parasites per host). Time 0 was considered as the day from when we were able to determine whether gammarids did actually harbour a parasite or not. Letters in the legend indicate significant differences between groups, with similar letters indicating no difference (odd-ratios from pairwise comparisons, *P* < 0.05)

Overall, control individuals survived better than infected ones, irrespective of the food treatment (Fig. 3a). Vouge parasites were slightly less lethal than Vair parasites in gammarids exposed to the standard food treatment (Fig. 3a), but not in those exposed to the deprived food treatment.

The deprived food treatment induced a significant decrease in the survival of control individuals, dropping to about half of that of individuals receiving the standard food treatment (Odd-Ratio from pairwise comparison, OR = 0.46, CI_95%_ = [0.34, 0.61], *P* <0.0001, Fig. 3a). This effect was also significant, but to a lower extent, in individuals exposed to Vouge parasites (OR = 0.75, CI_95%_ = [0.57, 0.99], *P* = 0.045, Fig. 3a), while no effect of food treatment was observed on the survival of individuals exposed to Vair parasites (*P* = 0.96, Fig. 3a).

Among infected individuals, a second Cox regression model (χ_2_ = 15.46, d.f. = 4, *P* = 0.004) confirmed that host survival was higher in individuals infected with Vouge parasites compared to those infected with Vair parasites (LR-χ_2_ = 7.67, d.f. = 1, *P* = 0.006). In addition, survival of individuals exposed to the standard food treatment was significantly higher than that of individuals exposed to the deprived food treatment (LR-χ_2_ = 4.50, d.f. = 1, *P* = 0.03). Finally, the number of parasites had a significant influence on survival (LR-χ_2_ = 7.93, d.f. = 2, *P* = 0.02, Fig. 3b), with a slightly better survival for gammarids harbouring a single parasite.

### Metabolic rate

**Fig. 4:**
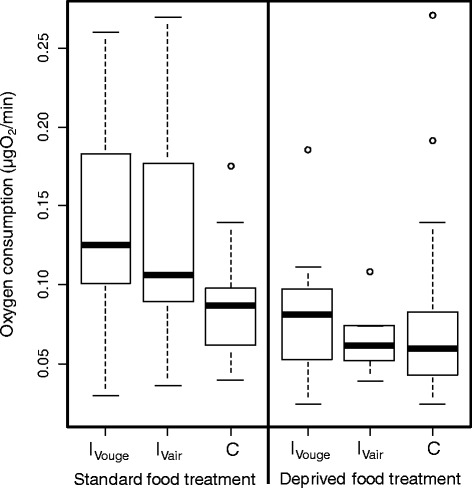
Hosts metabolism according to infection status and food treatment. Metabolic rate, expressed as oxygen consumption, of *Gammarus pulex* infected by parasites *Pomphorhynchus laevis* either from the Vouge (I_Vouge_) or the Vair populations (I_Vair_), or uninfected (control, C). Thick lines represent the medians, boxes represent the upper and lower quartiles, dotted lines represent the upper and lower deciles, and dots are outliers.

Oxygen consumption was significantly higher in infected individuals compared to control ones (ANOVA: F_-1,135_ = 11.10, *P* = 0.001; Fig. 4), and was lower in gammarids from the deprived food treatment compared to those from the standard food treatment (ANOVA: F_-1,135_ = 17.37, *P* <0.0001). Body mass of gammarids and all interactions were not significant and were removed from the model. Among infected individuals, a separate ANOVA indicated that the effect of food treatment was conserved (ANOVA: F_-1,55_ = 9.97, *P* = 0.003), whereas there was no effect of the population of origin of parasites (ANOVA: F_-1,55_ = 0.26, *P* = 0.61).

### Refuge use

**Fig. 5:**
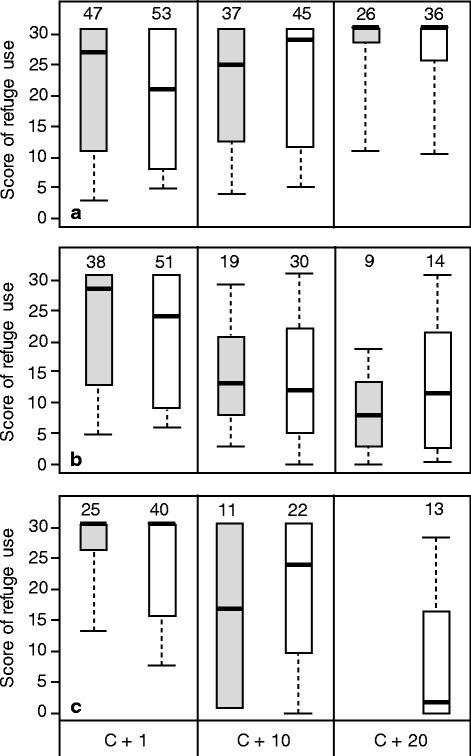
Host behaviour according to infection status and food treatment. Scores of refuge use for (**a**) control individuals; (**b**) individuals infected by parasites from the Vouge population; and (**c**) individuals infected by parasites from the Vair population. Grey plots represent groups who received the deprived food treatment and white plots stand for the standard food treatment. Scores are given for each of the three rounds: one day (C + 1), 10 days (C + 10) and 20 days (C + 20) after detection of cystacanth stages. Sample sizes are given above each plot. Thick lines represent the medians, the boxes represent the upper and lower quartiles and dotted lines represent the upper and lower deciles.

In the model based on the first two behavioural rounds, parasite intensity and food treatment had no effect on refuge use (results not presented) and were removed from the analysis. The remaining model showed that infection status, time (behavioural rounds) and the interaction between these two factors significantly influenced refuge use (Table 2, Fig. 5). Post-hoc pair-comparisons revealed that refuge use decreased between the first and the second round for gammarids infected with both *P. laevis* populations (Fig. 5b, c), whereas it remained stable in control individuals (Fig. 5a, pair-comparisons 1 and 2 in Table 2). The intensity of refuge use also differed between the two infected groups (Table 2, pair-comparison 3), with a more pronounced decrease in refuge use in gammarids infected by parasites from the Vouge river compared to those infected by parasites from the Vair river (Fig. 5b, c).

**Table 2:** Behavioural scores during the first two rounds for all individuals

Factor	Statistic	d.f.	P
**ANOVA TEST**			
Status	5.50	1.95	0.004
Round	21.13	1	<0.0001
Status x round	9.54	1.64	0.0002
**PAIR-COMPARISONS**			
***1) Vair-infected and Control individuals***			
Status	1.44	1	0.23
Round	4.53	1	0.03
Status x round	10.97	1	0.0009
***2) Vouge-infected and Control individuals***			
Status	4.90	1	0.03
Round	13.56	1	0.0002
Status x round	29.14	1	<0.0001
***3) Vair-infected and Vouge-infected individuals***			
Status	10.13	1	0.001
Round	29.95	1	<0.0001
Status x round	0.15	1	0.70

Results of the model from the nparLD R package, testing for the effects of status (Control, Vouge- and Vair-infected) and rounds of measurements on the scores of refuge use. Here, all individuals are considered regardless of their food treatment (not significant) but only the first two rounds are considered

The analysis of refuge use over the three series of behavioural tests, which was possible only for individuals from the standard food treatment (white labelling on Fig. 5), confirmed the strong interaction between infection status and rounds (Table 3). This interaction was due, as before, to the decrease vs. stability in refuge use between infected and control groups, respectively (Fig. 5, pair-comparisons 1 and 2 in Table 3). Refuge use also differed through time in infected individuals (Table 3, pair-comparison 3), with gammarids infected by parasites from the Vair river (Fig. 5c) decreasing their use of refuge during the third round, compared to gammarids infected with Vouge parasites (Fig. 5b).

**Table 3:** Behavioural scores during the three rounds for individuals from the standard food treatment

Factor	Statistic	d.f.	P
**ANOVA TEST**			
Status	6.35	1.95	0.002
Round	6.69	1.90	0.002
Status x round	13.05	3.41	<0.0001
**PAIR-COMPARISONS**			
***1) Vair-infected and Control individuals***			
Status	6.99	1	0.008
Round	2.99	1.97	0.051
Status x round	24.85	1.97	<0.0001
***2) Vouge-infected and Control individuals***			
Status	12.97	1	0.0003
Round	1.28	1.73	0.27
Status x round	13.53	1.73	<0.0001
***3) Vair-infected and Vouge-infected individuals***			
Status	0.63	1	0.43
Round	17.52	1.9	<0.0001
Status x round	3.90	1.9	0.02

Results of the model from the nparLD R package testing for the effects of status (Control, Vouge- and Vair-infected) and rounds of measurement on the scores of refuge use. Here, only individuals from the standard food treatment are considered and the analysis was conducted on the three behavioral rounds

Finally, all correlations between metabolic rate, survival and behavioural scores were non-significant (see Table 4).

**Table 4:** Correlations between metabolism, behaviour and survival

Factor	rho	n	P
**Metabolic rate vs behaviour**			
Vouge-infected	−0.034	31	0.86
Vair-infected	0.015	23	0.95
Controls	−0.13	78	0.27
**Survival vs behaviour**			
Vouge-infected	0.008	52	0.95
Vair-infected	−0.29	36	0.09
Controls	0.071	93	0.50

Spearman correlations between metabolic rate and behavioural scores, and between survival and behavioural scores (second behavioural round), for each infection status (individuals infected with the Vouge or the Vair population of parasites, and control individuals). When grouping the two infected groups, correlations were still not significant

## Discussion

Our results show that the experimental deprivation of host resources had significant effects on both host metabolism and survival. However, no consequence on the timing or the intensity of behavioural manipulation was observed.

### Effects of reduced host resources

Experimental deprivation of host resources, through a decrease in quality and quantity, led to several significant modifications in both parasites and hosts. First, food treatment had a significant effect on host metabolism. In accordance with Hervant *et al.* [44], the deprived diet induced a reduction in metabolic rate. This trend was conserved in infected individuals, while infection imposed an additional metabolic cost. Such an increase in metabolism has previously been reported in a crab parasitized by another acanthocephalan species [51] (but see Rumpus and Kennedy [52] for contradictory result). Second, the deprived food treatment induced a rise in the mortality rate of gammarids. Although this rise was observed for both control and infected individuals, the effect of food deprivation was higher in the former. Those two main changes in hosts suggest that the deprived diet was, as expected, responsible for a general decrease in host body condition. In addition to those changes, the deprived diet induced a negative effect on parasites from one of the two populations, in terms of intensity of infection, while other parameters of infection (i.e. prevalence and timing of development) remained unaltered.

However, and contrary to our expectations and the predictions made by the HERC hypothesis [12, 15, 16], the deprived diet, while affecting host body condition, did not affect the intensity nor the timing of parasite manipulation, independently of the population of origin of the parasites. Several explanations can be proposed to explain why food treatment did not affect the behaviour of infected hosts. First, contrary to what has been suggested [12, 15], behavioural alterations induced by parasites may not be a plastic, condition-dependant trait. Indeed, there was no correlation between either individual host survival or metabolic rate, and the intensity of behavioural manipulation, giving no evidence for any change in host exploitation strategy by parasites in terms of manipulation, following increased probability of host mortality. Second, differences induced by the two food treatments may not have been important enough to induce significant plastic changes. This is however unlikely because host metabolism, host survival and parasite intensity were all affected by the deprived food treatment. It is unlikely that such differences were due to a lower food consumption by infected hosts compared to uninfected ones, as Fielding *et al.* [53] showed that *Gammarus pulex* infected with another acanthocephalan parasite had similar feeding rates than controls, when they were fed with either leaves or dead chironomids. Third, resources may have been always sufficient to perform manipulation, such that food treatment would not influence host behaviour, particularly if the energetic cost of refuge use is low. However, the weaker effect of food treatment on infected host survival compared to controls suggests that parasites exploited more resources when they were available, thus leaving a minimum to their hosts, although those extra resources were not invested in host manipulation. Alternatively, host manipulation as a whole could be a phenomenon requiring less energy than previously thought, such that resources available would not be a significant parameter among those leading to the variations observed in the intensity of parasite manipulation (see Thomas *et al.* [3]). Finally, the dietary depletion experienced by individuals was based on only one type of food. Although we believe that reducing access to proteins and carotenoids (two major compounds present in chironomid larvae [54, 55]) constitutes a consequent dietary depletion, we cannot exclude the possibility that depletion of other types of resources would have different consequences on infection parameters and host manipulation by parasites.

The higher exploitation of resources observed in hosts fed with the standard diet implies that parasites may have allocated this extra-energy to other fitness traits. Parasites could first reach a higher success of infection. In this study, however, experimental infections were conducted before we manipulated food resources, such that hosts did not differ in body condition before the infection. It is then not surprising that no difference was observed in prevalence between treatments. In contrast, food deprivation had a negative effect on parasites intensity in one of the two populations. Beckage and Riddiford [56] also found that, in the lepidopteran species *Manduca sexta*, a lower number of hymenopteran parasites *Apanteles congregatus* would develop in hosts deprived from food. In the same way, other studies have shown that fewer parasites would develop if their hosts are starving [57–59]. Therefore, additional resources in the host may allow the coexistence of multiple parasites, probably reducing the competition that occurs among *P. laevis* sharing the same individual hosts [19]. Ultimately, this could be advantageous for the parasite because this would increase the probability of simultaneous transmission of several individuals, therefore increasing the probability of finding a mating partner in the definitive host [60].

An increase in the size of the parasites could also be a result of increased resources, leading to future beneficial effects, such as a better chance of establishment, and higher survival and fecundity in the definitive host [59, 61–63]. Several studies have shown that parasite size increases with host size [13, 64, 65], supporting a positive effect of higher levels of resources. Here, infection intensity significantly impacted the size of cystacanth larvae, confirming an effect of intra-host competition [19]. Although not significant, food treatment was however retained in the statistical model minimizing the AIC value, suggesting that this factor explains a part of the observed variance in parasite size, with cystacanth larvae being slightly smaller in the deprived food treatment.

### Effects of parasite population

Our study also provides further evidence for the implication of the population of origin of parasites on the variability observed in behavioural manipulation (see Thomas *et al.* [15] for a review), as well as on other parameters of infection [66]. Among all the parameters considered in this study, only time to reach the cystacanth stage and the change induced in the metabolic rate of hosts were independent of the parasite population. Consistent with Franceschi *et al.* [35], we found that parasites prevalence was different between the two populations of parasites studied here. In addition, the deprived food resources induced a decrease in intensity only in the Vair parasite population. These results suggest that parasites from the Vouge population already occupy the whole ecological niche offered by the host, even at lower resources, while those from the Vair population benefit from higher resources to establish. Differences in prevalence and intensity could then be due to a stronger resistance of the hosts against the Vair parasites.

Finally, consistent with several other studies [31, 35], different *P. laevis* populations differed in behavioural manipulation. Franceschi *et al.* [35] underlined that differences observed among several natural populations of parasites could be due to variation in the levels of resources in their environment. However, according to our results, it is more likely that those differences could be explained by other factors, such as intrinsic parameters of parasites population.

## Conclusions

While the experimental manipulation of the host food resources induced, as expected, significant differences in their body condition, our study suggests that resources are not likely to explain the observed inter-population variability in behavioural manipulation. However, overall, our results suggest that hosts in better condition may contribute to higher parasite success in populations, because they suffer less parasite virulence and can host more parasite larvae of slightly larger size.
